# Diagnostic Accuracy of the Swedish Version of the Multicultural Cognitive Examination for Cognitive Assessment in Swedish Memory Clinics

**DOI:** 10.3233/JAD-230998

**Published:** 2024-01-16

**Authors:** Rozita Torkpoor, Kristin Frolich, Elisabet Londos, T. Rune Nielsen

**Affiliations:** a Department of Clinical Sciences Malmö, Cognitive Disorder Research Unit, Lund University, Malmö, Sweden; b Memory Clinic, Skane University Hospital, Malmö, Sweden; c Department of Neurobiology, Division of Clinical Geriatrics, Care Sciences and Society, Karolinska Institutet, Stockholm, Sweden; d Danish Dementia Research Center, Copenhagen University Hospital, Rigshospitalet, Copenhagen, Denmark

**Keywords:** Alzheimer’s disease, assessment, cognition, dementia, ethnic minorities, immigrants, multicultural cognitive examination

## Abstract

**Background:**

Cognitive assessment for foreign-born individuals is suboptimal. The Multicultural Cognitive Examination (MCE) was developed for use in culturally, linguistically and educationally diverse populations. The MCE includes the Rowland Universal Dementia Assessment Scale (RUDAS) and performs assessment of memory, verbal fluency, and visuospatial function.

**Objective:**

To compare the psychometric properties of the Swedish version of the Multicultural Cognitive Examination (MCE-S) with the Swedish versions of the RUDAS (RUDAS-S), the Mini-Mental State Examination (MMSE-SR), and the Clock Drawing Test (CDT), and to explore the ability of the MCE-S test to differentiate patients with and without dementia in a multicultural population.

**Methods:**

117 outpatients at four memory clinics were tested using the MCE-S to complement the routine cognitive assessment.

**Results:**

Significant differences between patients with and without dementia were observed for all MCE-S components. There were significant differences between foreign-born and Swedish-born patients in the MMSE-SR, but not in the MCE-S or the RUDAS-S. The MCE-S, had good diagnostic performance for detecting dementia (AUC, 0.82), and was at least as good as the RUDAS-S alone (AUC, 0.79). The MCE-S also distinguished Alzheimer’s disease (AD) from non-AD dementia. Contrary to expectations, the MCE-S was also at least as good as the MMSE-SR among the Swedish-born patients.

**Conclusions:**

The MCE-S is adequate for detecting dementia in both foreign-born and Swedish-born populations. Based on the cultural diversity of general society, adapted cognitive tests that can be used for everyone are practical and beneficial for both patients and health-care professionals. Further studies are needed within primary care.

## INTRODUCTION

Similar to other European countries, the population of Sweden has become increasingly diverse in recent decades.

An increase in the number of older foreign-born individuals is expected in Europe because of demographic aging, which means an expected increase in the number of people with cognitive decline and dementia in this group [1–3]. This predicts that their aging profile will be correlated with a higher incidence of vascular and lifestyle risk factors [2, 4, 5].

The cognitive assessment of foreign-born individuals is associated with various challenges caused by communication difficulties, language barriers, and cultural background [6–11], the use of interpreters [1, 12–14], education background [2, 15, 16] and the tests used during the assessment [1, 2,16, 17].

Second-language effects are clear in most neuropsychological tests, and patients with a mother tongue other than Swedish can present lower scores in all Swedish-language-administered neuropsychological tests with a verbal component [18]. Previous studies have indicated that migrants may have lower odds of receiving a specific dementia diagnosis, lower use of dementia-specific drugs, and higher use of neuroleptics [19]; moreover, they may remain undiagnosed [20, 21], or be over-diagnosed if aged below 60 years and underdiagnosed if aged over 60 years [22].

The existing cognitive tests (e.g., the Mini-Mental State Examination (MMSE)) are not adjusted for multicultural populations and their scores are affected by language, cultural background, and education [1, 2]. The MMSE [23] is the tool most commonly used in memory clinics in Europe, and is also the preferred test for use in their foreign-born patients [22]. The need to develop adequate assessment methods for foreign-born patients is being increasingly recognized [1, 2, 11, 24, 25].

The Rowland Universal Dementia Assessment Scale (RUDAS) [26], which has been validated in many different countries, is a test with good diagnostic performance for detecting dementia across high-, low-, and middle-income settings and in samples with a lower and higher proportion of participants with no formal education [27]. The RUDAS was validated in Sweden and exhibited moderate-to-good diagnostic performance for detecting dementia [12]. Clinical experience and previous studies reveal a need for supplementary tests to the RUDAS for a more precise cognitive assessment of foreign-born individuals [12, 28, 29].

Several test batteries that can supplement the RUDAS have been developed to improve cognitive assessment among the multicultural populations of Europe, such as the Cross-cultural Neuropsychological Test Battery [28, 29], Cross-Cultural Dementia (CCD) [30] screening, and the Multicultural Cognitive Examination (MCE) [31]. The MCE cognitive screening instrument includes the RUDAS [26], the Recall of Pictures Test (RPT) [32], the Supermarket Fluency (SF) test [33], and the Clock Reading Test (CRT) [34].

### Aim

The aims of the study were to compare the psychometric properties of the Swedish version of the MCE (MCE-S) with the Swedish versions of the RUDAS (RUDAS-S), the MMSE (MMSE-SR), and the Clock Drawing Test (CDT), and to explore the ability of the MCE-S test to differentiate patients with dementia from those without dementia in a multicultural population at memory clinics in Sweden.

## MATERIAL AND METHODS

### Participants

All specialist memory clinics in southern Sweden were invited to participate in this prospective study. Four out of five clinics accepted the invitation and agreed to include at least 30 patients each. All health-care professionals at the memory clinics were given detailed information about the study trained by the research group (authors 1 and 2) in the administration of the MCE-S and in interpreter-mediated cognitive assessment. Thereafter the inclusion of patients was started.

Together with the regular invitation for the appointment at the memory clinics, an informative letter with the invitation to participate in the study was sent via regular post to potential participants. No exclusion criteria were formulated for participation in the study, to reflect the clinical patient population and give all individuals an equal opportunity to participate. The patients were referred for cognitive assessment and all had suspected cognitive impairment but no established diagnosis of dementia. When the patients came to the clinics for the cognitive assessment, they also declared whether they accepted or declined to participate in the study. Both the study information and consent forms were translated by translation agencies and were made available to patients who had not reached a reading level in Swedish. Verbal information and written study materials were provided to the patients at the visit. After the patients provided written consent, they were included in thestudy.

The inclusion began and continued based on practical circumstances at each clinic and took place between September 2018 and October 2019. According to the clinical activity, the four participating memory clinics started including patients at different time points. The patients were mostly referred from primary care or, in some cases, psychiatric and other specialist clinics.

Formal interpreters from the procured interpreting agency were available to all foreign-born patients, as needed. Although the cognitive assessment was performed using an interpreter for most foreign-born patients who participated in the study, 12 patients did not wish to have an interpreter because they were fluent in Swedish or would rather have the help of a relative. Of these 12 patients, nine originated from another Nordic or non-Nordic European country, one was from an Asian country, and two were from a South American country.

### Procedures

According to the national guidelines, the clinical diagnoses were based on physical, neurological, and psychiatric examinations; laboratory tests of blood samples; brain imaging; cognitive tests; and interviews with relatives [35]. The routine cognitive tests included the MMSE-SR and the CDT. In some patients, Cube copying and lumbar puncture were performed. The MMSE-SR and the CDT were used for diagnosis, whereas the MCE-S was not part of the diagnostic procedure.

In the present study, the MMSE-SR, the CDT [36], with a range of 0–5, and the MCE-S, including the RUDAS-S, were used to assess cognitive impairment. To evaluate the instrumental activities of daily living (IADL), the Functional Assessment Questionnaire (FAQ) [37], with a range of 0–30 points, was used. A score of ≥9 points, or dependence in three or more activities, indicated impaired IADL. To identify a depressive component, the 20-item Swedish version of the Geriatric Depression Scale (GDS-20) with a cutoff of ≥6 points was used [38].

### Multicultural cognitive examination

The MCE [31] is a brief cross-cultural cognitive screening instrument with a total score of 100 points. The MCE does not require any specialized training and may be useful for the classification of mild dementia or dementia subtypes. The MCE is a comprehensive combination of sensitive instruments and comprises four parts that assess separate cognitive functions, respectively. The test includes the RUDAS for the assessment of general cognitive functioning [26], the Recall of Pictures Test (RPT) for memory [32], the Supermarket Fluency (SF) test for verbal fluency [33], and the CRT for visuospatial function [34] (Table 1).

**Table 1:** The Multicultural Cognitive Examination

Cognitive domain	Item	Points
General Cognitive Function	RUDAS (memory and memory recall, visuospatial orientation, praxis, visuoconstructional drawing, judgment, and language)	30
Memory^*^	Recall of Pictures Test (immediate recall, delayed recall, and recognition of 10 pictures)	30
Language/executive	Supermarket Fluency (the number of different supermarket items produced in 1 min)	28
Visuospatial	Clock Reading Test (reading of the time on 12 clock faces)	12
Total score	/100

^*^Immediate recall is the mean score of three learning experiences. Delayed recall is the number of pictures that the participant can recall after an interference interval; score, 0–10. Recognition is the number of pictures the participant can recognize among 10 other pictures, subtracted by the number of false-positive answers; score, 0–10.

Previously, we translated and back-translated the RUDAS into Swedish [12] according to established principles [39]. The same procedures were used to prepare the MCE-S.

After the study, three or four health-care professionals, nurses or physicians, from each participating clinic shared their experiences of the tests in three focus-group interviews.

### Diagnoses

All diagnoses were established according to the International Classification of Diseases (ICD-10) diagnostic system [40] and included etiological diagnosis and level of cognitive impairment. The cognitive impairment was classified as dementia, mild cognitive disorder corresponding to mild cognitive impairment (MCI), and unspecified symptoms and signs involving cognitive functions and awareness, corresponding to subjective cognitive impairment (SCI). The McKeith criteria were used for the diagnosis of dementia with Lewy bodies (DLB) in the memory clinics [41]. In Sweden, the code F02.8 G31.8 is used for DLB, as it is not specified in ICD-10. The diagnoses were established via consensus in joint diagnosis rounds in which several physicians and other health-care personnel from the different memory clinics participated.

### Ethics

The local ethics committee of Lund University approved the study (2016/292 and 2018/109). Informed consent was obtained from all participants.

### Statistical analysis

Descriptive statistics were applied to compare demographic and clinical characteristics. Student’s *t* test was used to compare group means of continuous, normally distributed variables (e.g., age), whereas the Mann–Whitney *U* test was used to compare continuous, nonnormally distributed variables and variables with uneven scaling properties (e.g., cognitive test results, GDS, and FAQ). Fisher’s exact test (e.g., sex) and Pearson’s χ^2^ test were used to test differences in the distribution of categorical variables. Spearman’s correlations were used for analyses between continuous variables (age, years of education, MMSE-SR, CDT, RUDAS-S, MCE-S, and FAQ). Binary logistic regression analyses of the MCE-S were performed to investigate the probability of dementia adjusted by age, sex, years of education, and foreign-/Swedish-born status. Missing data in years of education were substituted by multiple imputations using all test data, age, and education.

The effect size was calculated as *η*^2^ for ANOVA (0.01, small effect; 0.06, medium effect; and 0.14, large effect), as well as *r* for the Mann–Whitney *U* test (<0.3, small effect; 0.3–0.5, medium effect; and >0.5, large effect).

Receiver operating characteristic (ROC) curve analyses were applied to test the ability of the MMSE-SR, CDT, RUDAS-S, and MCE-S to identify dementia, as the criterion standard. No other covariates were used. An area under the ROC curve (AUC) between 0.9 and 1.0 is considered excellent, between 0.8 and 0.9 is considered good, between 0.7 and 0.8 is considered fair, between 0.6 and 0.7 is considered poor, and between 0.5 and 0.6 is considered a failure [42].

The Clinical Calculator 1 from the Vassar Stats website (www.vassarstats.net/clin1.html) was used to calculate sensitivity, specificity, positive predictive value (PPV), negative predictive value (NPV), and likelihood ratio (LR)+, and LR–with 95% confidence intervals (CIs) at different cutoff points. All other analyses were performed using IBM SPSS Statistics for Windows (version 27; IBM Corp., Armonk, NY, USA). Significance was set at *p* < 0.05.

## RESULTS

### Participant characteristics

**Fig. 1:**
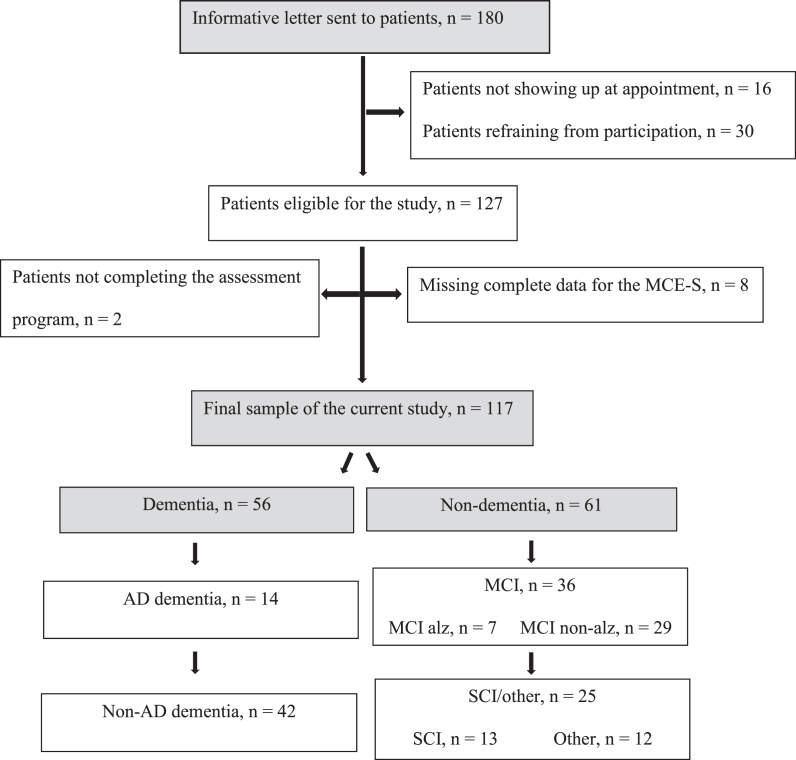
Flow chart: Participants and clinical diagnoses.

Of the 180 patients who received the informative letter, 127 agreed to participate in the study and 125 received a diagnosis. However, as not all patients underwent the MCE-S test in its entirety, data were analyzed based on the 117 individuals who answered all questions included in the MCE-S (Fig. 1).

Eighty-seven patients were born in Sweden and 30 were born elsewhere: five originated from another Nordic country (Denmark, Finland, or Norway), 12 from an Eastern European country (Bosnia, Poland, former Yugoslavia, Kosovo, Makedonia, or Belarus), six from a Middle Eastern country (Iran, Iraq, Lebanon, or Palestine), three from an Asian country (Thailand or Taiwan), three from a South American country (Brazil or Chile), and one was from an African country (Cameroon). According to the World Bank Group’s country classification, which indicates socioeconomic position, 10 participants were classified as coming from a high-, 16 from an upper-middle-, and four from a lower-middle income country. Foreign-born participants had lived in Sweden for 1–55 years (mean, 30 years).

The comparison of foreign-born and Swedish-born patients revealed the absence of significant differences in age (68.8±13.0 years; range, 43–88 versus 70.8±11.8 years; range, 30–92 years) or sex (19 females/11 males versus 43 females/44 males). Moreover, compared with Swedish-born patients, foreign-born participants had fewer years of education (9.6±4.0 years; range, 1–17 years versus 11.3±3.6 years; range, 6–20 years; *p* = 0.05).

The comparison of foreign-born and Swedish-born patients showed that the mean MMSE-SR score was significantly lower in the former (19.8±5.5 versus 24.8±4.8, *U* = 613.5, *p* < 0.001, *r* = 0.40), followed by the score on the CDT (2.0±1.8 versus 3.6,±1.5, *U* = 952.0, *p* = 0.05, *r* = 0.18). In the MCE-S there was a significant difference in the SF task (12.0±5.8 versus 15.0±6.6, *U* = 941.0, *p* = 0.02, *r* = 0.21), but not in any of the remaining MCE-S measures, including total score (63.0±18.0 versus 67.0±17.3) and RUDAS-S score (23.1±4.6 versus 22.6±4.5). Mean time in months from first consultation to diagnosis was 5.1±1.7 for the foreign-born and 5.3±3.1 for the Swedish-born patients. Prevalence of dementia diagnoses did not differ between the foreign-born (50%) and Swedish-born (47%) patients. Figure 1 reports the distribution of the diagnoses.

Fifty-six patients were diagnosed with dementia, 36 with MCI (F067) and 25 with SCI (13 with memory-related subjective symptoms (R418A) and 12 with other diagnoses, such as depression, posttraumatic stress disorder (PTSD), burnout syndrome, fibromyalgia, and neurological disorders such as dysphasia and parkinsonism). Among those with dementia (*n* = 56), 14 patients had Alzheimer’s disease (AD), 10 had vascular dementia (VaD), 18 had mixed AD/VaD, four had DLB/Parkinson’s disease dementia (PDD), and five had frontotemporal dementia (FTD). Five patients were diagnosed with nonspecific dementia. Among those with MCI, seven patients had positive AD CSF biomarkers. The distribution of diagnoses did not differ significantly between foreign-born and Swedish-bornpatients.

The demographic and cognitive characteristics of the patients with a diagnosis of clinical dementia, MCI, and SCI are summarized in Table 2. Significant group differences were detected for FAQ-IADL, GDS-20, and all cognitive measures. Patients with dementia had the lowest scores across all cognitive measures, followed by patients with MCI and those with SCI.

**Table 2:** Demographic characteristics, test results and cognitive diagnoses of the study population (N = 117)

Characteristic	Total	Dementia	Non-Dementia		*p*
			MCI	SCI
N	117	56	36	25
Age, y, m (SD)	70.3(12.0)	76.5 (6.6)	68.7 (12.0)	58.7 (12.7)	<0.001
Sex, Female, (N) %	(62) 53%	(32) 57%	(16) 44.4%	(14) 56%	0.46^a^
Years of education	10.9 (3.8)	10.3 (3.7)	10.9 (3.8)	12.1 (3.8)	0.31
Foreign-Born, (N) %	(30) 26%	(15) 27%	(7) 19%	(8) 32%	0.52^a^
Swedish-Born, (N) %	(87) 74%	(41) 73%	(29) 81%	(17) 68%
FAQ, m (SD), N = 105	12.3 (7.9)	15.5 (7.5)	9.8 (6.5)	8.2 (8.0)	<0.001
GDS, m (SD), N = 110	5.2 (4.09)	4.4 (3.3)	4.8 (3.6)	7.6 (5.2)	0.004
MMSE, m (SD)	23.6 (5.4)	21.0 (5.7)	25.2 (3.9)	26.8 (4.0)	<0.001
CDT, m (SD), N = 115	3.5 (1.6)	3.0 (1.7)	3.9 (1.4)	4.1 (1.2)	0.004
MCE, m (SD)	66 (17.5)	55.9 (13.8)	70.5 (15.8)	82.1 (11.5)	<0.001
RUDAS	22.7 (4.5)	20.4 (3.9)	23.4 (3.9)	27.1 (2.6)	<0.001
RPT, Immediate Recall	5.6 (2.4)	4.3 (2.3)	6.0 (1.8)	7.8 (1.6)	<0.001
RPT, Delayed Recall	5.3 (3.2)	3.4 (2.7)	6.1 (2.8)	8.12 (2.3)	<0.001
RPT, Recognition	8.8 (2.0)	8.1 (2.4)	9.2 (1.7)	9.7 (0.6)	0.001
SF	14.2 (6.5)	11.5 (5.2)	15.7 (6.6)	18.4 (6.3)	<0.001
CRT	9.8 (3.0)	8.6 (3.6)	10.9 (1.6)	10.9 (2.0)	<0.001

Abbreviations: FAQ, Functional Assessment Questionnaire; GDS, Geriatric Depression Scale; MMSE, Mini Mental Scale Examination; CDT, Clock Drawing Test; MCE, Multicultural Cognitive Examination; RUDAS, Rowland Universal Dementia Assessment Scale; RPT, Recall of Pictures Test; SF, Supermarket Fluency; CRT, Clock Reading Test; SD, standard deviation; M, mean; N, number. Non-dementia included MCI, SCI, depression, posttraumatic stress disorder (PTSD), burnout syndrome, fibromyalgia, and neurological disorders (such as dysphasia and parkinsonism). a =  χ^2^.

Significant differences between patients with and without dementia were detected for all MCE components, with a large effect size (RUDAS, RPT, SF, CRT, *η*^2^ = 0.10–0.29). The mean MCE-S score was 55.9±13.8 in patients with dementia, whereas in non-dementia cases it was as follows: MCI, 70.5±15.8; and SCI, 82.1±11.5 (F (2, 114) = 32.9, *p* < 0.001, *η*^2^ = 0.37).

### Psychometric properties

The internal consistency of the MCE-S was acceptable, with a Cronbach alpha of 0.78 (a value >0.70 is considered acceptable) [43]. The MCE-S total score was strongly and significantly correlated with all MCE-S subcomponents (*p* < 0.001) in the whole group, as follows: RUDAS-S (*r* = 0.846, *p* < 0.001), SF (*r* = 0.825, *p* < 0.001), RPT immediate recall (*r* = 0.801, *p* < 0.001), RPT delayed recall (*r* = 0.789, *p* < 0.001), RPT recognition (*r* = 0.611, *p* < 0.001), and CRT (*r* = 0.686, *p* < 0.001). Strong significant correlations were observed between the MCE-S total score and MMSE-SR (*r* = 0.726, *p* < 0.001) and CDT (*r* = 0.496, *p* < 0.001).

### Diagnostic accuracy

Data on diagnostic accuracy pertaining to the ability of the MCE-S to differentiate patients with from those without dementia are presented in Table 3.

**Table 3:** Diagnostic accuracy of the MCE at different cutoff scores (N = 117)

Cutoff score	YI	Sensitivity (95% CI)	Specificity (95% CI)	PPV (95% CI)	NPV (95% CI)	LR+ (95% CI)	LR–(95% CI)	*A%
<68/100	50	0.78 (0.65–0.88)	0.72 (0.59–0.82)	0.72 (0.59–0.82)	0.78 (0.65–0.88)	2.59 (1.68–3.99)	0.27 (0.16–0.45)	0.75
<69/100	52	0.80 (0.67–0.89)	0.72 (0.59–0.82)	0.72 (0.59–0.83)	0.80 (0.67–0.89)	2.65 (1.72–4.08)	0.25 (0.15–0.43)	0.76
<70^†^/100	58	0.84 (0.71–0.92)	0.74 (0.61–0.84)	0.75 (0.62–0.84)	0.83 (0.70–0.92)	2.94 (1.88–4.59)	0.2 (0.11–0.36)	0.79
<71/100	53	0.86 (0.73–0.93)	0.67 (0.54–0.78)	0.70 (0.49–0.67)	0.84 (0.70–0.92)	2.4 (1.61–3.58)	0.19 (0.10–0.37)	0.76
<72/100	54	0.89 (0.77–0.95)	0.65 (0.52–0.77)	0.70 (0.58–0.80)	0.87 (0.73–0.94)	2.38 (1.61–3.51)	0.15 (0.07–0.32)	0.77

YI, Youden Index = Sensitivity (%) + Specificity (%) –100; CI, confidence interval; PPV, positive predictive value; NPV, negative predictive value; LR+, positive likelihood ratio = Sensitivity / (1 –Specificity); LR–, negative likelihood ratio = (1 –Sensitivity) / Specificity; *A, Accuracy = true positive + true negative / all cases, LR+ and LR–weighted for prevalence; ^†^optimal cutoff value. At the optimal cutoff of <70/100 points, the MCE-S had a sensitivity of 0.84 and a specificity of 0.74. Twenty-three patients with a diagnosis of dementia (41%) had scores above the cutoff for cognitive impairment on the MMSE-SR, nine patients (16%) on the MCE-S and five patients (9%) on the RUDAS-S. Among the 23 patients with dementia, 16 (70%) had MCE-S scores <70 and 14 (61%) had RUDAS-S scores <25, and all were Swedish-born individuals.

**Fig. 2:**
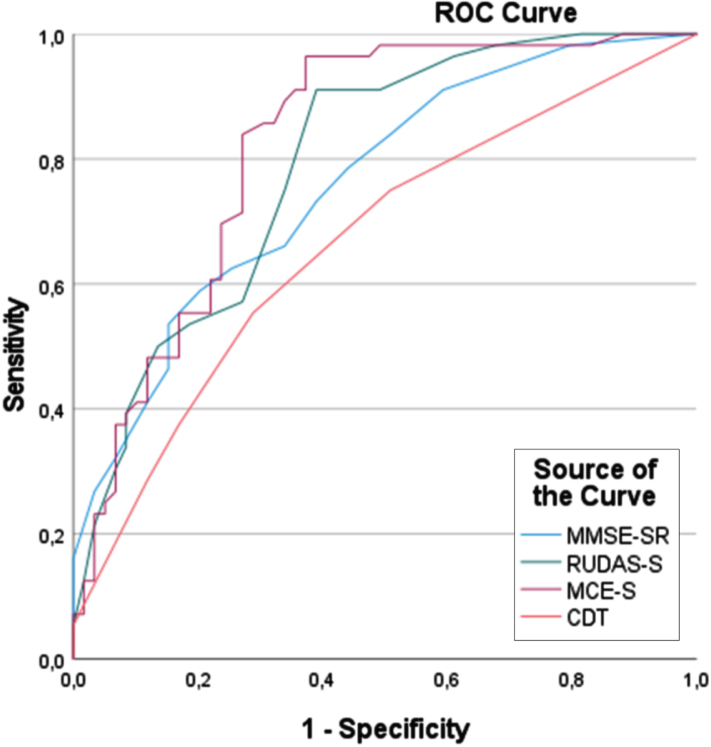
ROC curve analysis of the MCE-S, RUDAS-S, MMSE-S, and CDT for detecting dementia (N = 115). MCE-S, Swedish version of the Multicultural Cognitive Examination; RUDAS-S, Swedish version of the Rowland Universal Dementia Assessment Scale; MMSE-SR, Swedish version of the Mini Mental Scale Examination; CDT, Clock Drawing Test.

ROC curve analyses were used to compare the accuracy for the diagnosis of dementia of the cognitive tests, i.e., MCE-S, RUDAS-S, MMSE-SR, and CDT (Fig. 2). The analyses revealed that the MCE-S (AUC, 0.82) was at least as good as the RUDAS-S (AUC, 0.79), MMSE-SR (AUC, 0.76), and CDT (AUC, 0.67) in distinguishing patients with from those without dementia.

Moreover, an ROC curve analysis revealed the absence of differences in the diagnostic accuracy of the MCE-S between the foreign-born (AUC, 0.82) and Swedish-born (AUC, 0.83) patients.

The repetition of the analyses in a subgroup of patients with dementia (*n* = 56) and SCI (*n* = 25) yielded AUC values of 0.92 for the MCE-S and RUDAS-S, 0.81 for the MMSE-SR, and 0.70 for the CDT.

### Comparison of AD dementia and non-AD dementia

Among the 56 patients with dementia, 14 were diagnosed with AD dementia and 42 with non-AD dementia. The comparison of the AD and non-AD dementia groups using the different parts of the MCE-S showed that patients with AD dementia had significantly lower scores on RPT delayed recall (*U* = 174.5, *p* = 0.02, *r* = 0.31), whereas there were trends toward significance on RPT immediate recall (*p* = 0.06) and MCE-S total score (*p* = 0.08) (Table 4).

**Table 4:** Comparison of the mean scores on the MCE-S and its sub-components among patients with AD and non-AD dementia

MCE -S and MCE-S sub-component	AD, N = 14	Non-AD, N = 42	*p*
	Mean (SD)	Mean (SD)
MCE-S total score	50.4 (11.8)	57.8 (14.0)	0.08
RUDAS-S	19.1 (3.7)	20.8 (3.9)	0.17
RPT, immediate recall	3.3 (1.7)	4.6 (2.4)	0.06
RPT, delayed recall	2.1 (2.3)	3.9 (2.6)	0.02^a^
RPT, recognition	7.4 (2.9)	8.4 (2.3)	0.19
SF	10.9 (4.2)	11.7 (5.5)	0.61
CRT	7.3 (4.1)	9.0 (3.4)	0.14

AD, Alzheimer’s disease; MCE-S, Swedish version of the Multicultural Cognitive Examination; RUDAS-S, Swedish version of the Rowland Universal Dementia Assessment Scale; RPT, Recall of Pictures Test; SF, Supermarket Fluency; CRT, Clock Reading Test; n, number; SD, standard deviation. ^a^Mann–Whitney *U* test.

### Effect of demographic variables on the diagnosis of dementia

A binary logistic regression analysis of MCE-S was conducted to evaluate the effect of the demographic variables on the ability of this instrument to classify dementia. In this regression model, the probability of dementia was significantly predicted by age (*p* = 0.001) and the MCE-S score (*p* < 0.001), but not by sex, years of education, or foreign-/Swedish-born status.

### Face validity

The health-care professionals who participated in this study reported that the MCE-S was a test that was easy to administer and to use through an interpreter. Namely, the test contained no questions that were perceived as difficult to ask through an interpreter compared with the MMSE-SR. A recurrent example of this was the repetition question where the patient must say ‘no ifs, buts, or whys’. There has always been uncertainty in asking that question, as it has usually been problematic, both for the interpreter to transmit it and for the patient to understand it.

## DISCUSSION

In this validation study of the MCE-S in a mixed population of foreign-born and Swedish-born outpatients, we showed that the MCE-S had good diagnostic performance for detecting dementia (AUC, 0.82) and was at least as good as the RUDAS alone. The MCE-S as a screening instrument was also useful for the classification of milder cognitive symptoms, i.e., for differentiating MCI from SCI. The MCE-S with its various subcomponents may be an opportunity to assess the cognitive profile and, to a certain extent, guide the specific dementia diagnostics. These results confirmed a previous validation study of the MCE [31]. In contrast to our expectation, the MCE-S functioned at least as good among the Swedish-born patients compared with the MMSE-SR.

Previous studies have highlighted the importance of cognitive tests that are sensitive for identifying dementia without the effects of language, culture, and educational background [17, 24]. Although the RUDAS-S is currently recommended in Sweden, the extension of complementary tests for cognitive examination, such as the remaining subcomponents of the MCE, is warranted. Our results showed that the MCE-S was not affected by factors such as language and educational background, or whether the patient was born in Sweden or elsewhere. This was illustrated by lower mean MMSE-SR and CDT scores among foreign-born participants, regardless of whether they had a diagnosis of dementia. This difference was not observed in the MCE-S total scores or in most parts of the MCE-S test. By adding different test parts, which specifically assess the patient’s memory, language, and visuospatial function, to the RUDAS-S, the MCE-S had a slightly higher diagnostic accuracy than the RUDAS-S alone and the MMSE-SR. In addition, in line with previous research, the RPT delayed recall component of the MCE-S was not affected by education and foreign-born or Swedish-born status, and was highly sensitive to dementia [28, 31, 32, 44]. The comparison of patients with AD and non-AD dementia revealed that patients with AD dementia were more impaired in the memory domain, especially in delayed recall. This despite the suspicion that the non-AD group probably contains individuals with a mixed pathology including AD. Impairment in delayed recall was consistent with that reported previously [45] and supports earlier claims that the MCE-S may be useful for identifying profiles of cognitive impairment that differentiate patient groups with AD and non-AD dementia from each other [31]. This finding is especially interesting because early detection of AD can be decisive for patients and for the administration of treatment and support (see Supplementary Material).

This study also showed that CDT, but not CRT, was affected by education and the foreign-born or Swedish-born status, which agreed with Nielsen et al. [29]. Moreover, Maestri et al. [46] suggested that the application of the CDT in a multicultural population should be performed with care.

Our study yielded an AUC of 0.82 and a cutoff score <70 for the MCE-S. In the regression analysis, the ability of the MCE-S (and all subcomponents) to predict dementia was affected by age exclusively, which may indicate that the diagnostic properties of the MCE-S are relatively unbiased regarding the educational background and whether the patient is a foreign-born or Swedish-born individual. The high internal correlation observed between the different parts of the MCE-S strengthens the ability of the test to distinguish different degrees of cognitive impairment (SCI, MCI, and dementia). Our results support a previous validation study of MCE [31], which yielded the exact same cutoff scores for the MCE as those reported here, with an AUC >0.90. Nielsen et al. [31] studied patients with dementia and healthy controls, whereas our population consisted of patients with dementia, MCI, SCI, and other diagnoses who were undergoing investigations at memory clinics, which could explain the different AUCs obtained. The present study expanded the findings of Nielsen et al. on early and mild dementia by also analyzing milder cognitive impairment levels, such as MCI and SCI [31].

Although the diagnoses were established in specialist clinics and were the gold standard for our study, the diagnoses mentioned herein can still be questioned, e.g., the MCI diagnosis. We noticed that many of the patients received MCI diagnosis already after the first contact with the specialist clinics (due to local traditions) and that, eventually, many of these diagnoses were changed to other conclusions after the diagnostic rounds. But we still suspect because of the test results that there may be some patients with MCI who should have received a diagnosis of dementia, i.e., that MCI was over-diagnosed, which may have affected our analyses.

The experience of the participating health-care professionals regarding the use of the MCE-S supports previous studies, which applied the test in more than 20 languages and reported that the test is easy to administer both with and without an interpreter and without having to change the content of the test [29]. By contrast, the MMSE, which is a highly verbal screening test [47], was perceived as being difficult to use among foreign-born patients or through an interpreter.

### Strengths and limitations

The strengths of the present study were that there were no differences between the foreign-born and Swedish-born groups in terms of sex and age. There was only a trend toward a significant difference in years of education between the foreign-born and Swedish-born patients, whereas we cannot explain the differences in the results of the conventional cognitive tests, such as the MMSE-S and CDT, as solely being related to education. The time lived in Sweden of the participants varied between 1 and 55 years, with an average of 30 years. We propose that having a different country of birth and a different cultural background (versus Sweden) was a disadvantage for the foreign-born participants, despite the use of an interpreter. This speaks against the use of customary (culture- and education-dependent) tests among foreign individuals who have lived for a long time in a foreign country. One of the limitations of the study was that a measurement of acculturation was not performed, as a lower degree of acculturation could be related to poorer performance in cognitive testing [48–50], specifically in tests of cognitive speed and executive ability [51]. This is important to consider for future studies of MCE subcomponents in relation to different dementia disorders.

To reflect real life, no exclusion criteria for participation were set in this study. One of the advantages of the study was that it included patients with different mother tongues and cultural backgrounds, which were representative of the most common foreign-born groups in Sweden [52], as well as Swedish patients. Interestingly, the study showed that the MCE-S test identified Swedish patients with dementia more accurately than the MMSE-SR.

The setting of the present study was specialized memory clinics, and most of the patients were referred from primary care. Therefore, the MCE-S test could probably be used in primary care.

Conversely, the small number of foreign-born patients included in the study was one of its drawbacks. This might reflect the lower number of foreign-born patients seeking care for cognitive disorders [1] and that the proportion of foreign-born in the different catchment areas for the memory clinics varies, which in turn affects the number of foreign-born in the study.

This study was carried out in different specialist clinics with different routines and expertise. Even if the diagnoses were established based on consensus, we are not entirely sure that they were completely correct, especially among foreign-born patients, who tend to be misdiagnosed [8, 10]. The fact that an interpreter was used in some cases may have partially affected the patient’s test results, as shown in previous studies [12, 13, 53].

Another study limitation was that the results of the MMSE-SR were used to establish the diagnosis of dementia. This may have affected the psychometric properties of the MMSE-SR because of circular reasoning. Moreover, the clinical dementia diagnosis was used as a gold standard, and use of a separate measure of cognitive impairment, such as the clinical dementia rating scale, would have been advantageous [54].

In summary, this validation study of the MCE-S was used for the cognitive assessment of both foreign-born (from 18 different countries) and Swedish-born patients. The MCE-S exhibited good accuracy, at least as good as that of the RUDAS-S alone, for identifying dementia in a Swedish context. Furthermore, the test had the ability to distinguish AD from non-AD dementia, as well as different cognitive levels (dementia, MCI, and SCI) from each other, which indicates good test properties as a screening instrument for the early diagnosis of cognitive impairment.

In conclusion, this MCE-S validation study showed that this test has good properties for use in both foreign-born and Swedish-born populations. Based on the cultural diversity of general society, adapted cognitive tests that are sensitive for identifying dementia and can be used for everyone are practical and beneficial for both the patients and the health-care staff who perform the investigations. Further studies are needed with larger patient groups, for people who are illiterate or have few years of schooling and also within a primary care setting.

## Supplementary Material

Supplementary Material

## Data Availability

The data supporting the findings of this study are available upon request from the corresponding author. The data are not publicly available due to privacy or ethical restrictions.

## References

[1] Gove D , Nielsen RT , Smits C , Plejert C , Rauf M A , Parveen S , Jaakson S , Golan-Shemesh D , Lahav D , Kaur R , Herz MK , Monsees J , Thyrian JR , Georges J (2021) The challenges of achieving timely diagnosis and culturally appropriate care of people with dementia from minority ethnic groups in Europe. Int J Geriatr Psychiatry 36, 1823–1828. 34378237 10.1002/gps.5614 PMC9291493

[2] Nielsen TR (2022) Cognitive assessment in culturally, linguistically, and educationally diverse older populations in Europe. Am J Alzheimers Dis Other Demen 37, 1–8. 10.1177/15333175221117006 PMC10581111 36325840

[3] Prince M , Bryce R , Albanese E , Wimo A , Ribeiro W , Ferri CP (2013) The global prevalence of dementia: A systematic review and metaanalysis. Alzheimers Dement 9, 63–75. 23305823 10.1016/j.jalz.2012.11.007

[4] Adelman S , Blanchard M , Livingston G (2009) A systematic review of the prevalence and covariates of dementia or relative cognitive impairment in the older African–Caribbean population in Britain. Int J Geriatr Psychiatry 24, 657–665. 19235788 10.1002/gps.2186

[5] Livingston G , Leavey G , Kitchen G , Manela M , Sembhi S , Katona C (2001) Mental health of migrant elders— The Islington study. Br J Psychiatry 179, 361–366. 11581119 10.1192/bjp.179.4.361

[6] Brown S , Livingston G , Mukadam N (2021) A national memory clinic survey to assess provision for people from diverse ethnic backgrounds in England and Wales. Int J Environ Res Publ Health 18, 1456. 10.3390/ijerph18041456 PMC7913949 33557171

[7] Schoenmakers B , Robben T (2021) Barriers in screening for dementia in elderly migrants in primary care and the use of the Rowland Universal Dementia Assessment Scale. A mixed cross-sectional and qualitative study. Eur J Gen Pract 27, 45–50. 33928835 10.1080/13814788.2021.1913116 PMC8816395

[8] Tillmann J , Just J , Schnakenberg R , Weckbecker K , Weltermann B , Münster E (2019) Challenges in diagnosing dementia in patients with a migrant background— a cross-sectional study among German general practitioners. BMC Fam Pract 20, 34. 30803438 10.1186/s12875-019-0920-0 PMC6388491

[9] Sagbakken M , Storstein Spilker R , Nielsen TR (2018) Dementia and immigrant groups: A qualitative study of challenges related to identifying, assessing, and diagnosing dementia. BMC Health Serv Res 18, 910. 30497459 10.1186/s12913-018-3720-7 PMC6267848

[10] Nielsen TR , Vogel A , Riepe MW , de Mendonca A , Rodriguez G , Nobili F , Gade A , Waldemar G (2011) Assessment of dementia in ethnic minority patients in Europe: A European Alzheimer’s Disease Consortium survey. Int Psychogeriatr 23, 86–95. 20602861 10.1017/S1041610210000955

[11] Nielsen TR , Antelius E , Storstein Spilker R , Torkpoor R , Toresson H , Plejert C (2015) Dementia care for people from ethnic minorities: A Nordic perspective. Int J Geriatr Psychiatry 20, 217–222. 10.1002/gps.4206 25639833

[12] Torkpoor R , Frolich K , Nielsen TR , Londos E (2022) Diagnostic accuracy of the Swedish version of the Rowland Universal Dementia Assessment Scale (RUDAS-S) for multicultural cognitive screening in Swedish memory clinics. J Alzheimers Dis 89, 865–876. 35964182 10.3233/JAD-220233 PMC9535584

[13] Haralambous B , Tinney J , LoGiudice D , Meng Lee S , Lin X (2018) Interpreter-mediated cognitive assessment: Who wins and who loses? Clin Gerontol 41, 227–236. 29240549 10.1080/07317115.2017.1398798

[14] Plejert C , Antelius E , Yazdanpanah M , Nielsen TR (2015) “There’s a letter called ef”: On challenges and repair in interpreter-mediated tests of cognitive functioning in dementia evaluations: A case study. J Cross-Cult Gerontol 30, 163–187. 25982531 10.1007/s10823-015-9262-0

[15] Ardila A , Bertolucci PH , Braga LW , Castro-Caldas A , Judd T , Kosmidis MH , Matute E , Nitrini R , Ostrosky-Solis F , Rosselli M (2010) Illiteracy: The neuropsychology of cognition without reading. Arch Clin Neuropsychol 25, 689–712. 21075867 10.1093/arclin/acq079

[16] Nielsen TR , Jørgensen K (2013) Visuoconstructional abilities in cognitively healthy illiterate Turkish immigrants: A quantitative and qualitative investigation. Clin Neuropsychol 27, 681–692. 23379740 10.1080/13854046.2013.767379

[17] Franzen S , van den Berg E , Goudsmit M , Jurgens CK , van de Wiel L , Kalkisim Y , Uysal-Bozkir Ö , Ayhan Y , Nielsen TR , Papma JM (2020) A systematic review of neuropsychological tests for the assessment of dementia in non-Western, low-educated or illiterate populations. J Int Neuropsychol Soc 26, 331–351. 31511111 10.1017/S1355617719000894

[18] Stålhammar J , Hellström P , Eckerström C , Anders Wallin A (2022) Neuropsychological test performance among native and non-native Swedes: Second language effects. Arch Clin Neuropsychol 37, 826–838. 32722802 10.1093/arclin/acaa043 PMC9113439

[19] Lindgren E , Sörenson J , Wattmo C , Kåreholt I , Nägga K (2021) Differences in dementia care between Swedish-born and foreign-born from countries with different country level socioeconomic position: A nationwide register-based study. J Alzheimer Dis 84, 1363–1371. 10.3233/JAD-210734 PMC8673530 34657886

[20] Franco Y , Choi EY (2020) The relationship between immigrant statusand undiagnosed dementia: The role of limited English proficiency. J Immigr Minor Health 22, 914–922. 31893329 10.1007/s10903-019-00963-w

[21] Selten JP , Termorshuizen F , van Sonsbeek M , Bogers J , Schmand B (2020) Migration and dementia: A meta-analysis of epidemiological studies in Europe. Psychol Med 51, 1838–1845. 32264980 10.1017/S0033291720000586 PMC8381287

[22] Nielsen TR , Vogel A , Phung TK , Gade A , Waldemar G (2011) Over- and under-diagnosis of dementia in ethnic minorities: A nationwide register-based study. Int J Geriatr Psychiatry 26, 1128–1135. 21194100 10.1002/gps.2650

[23] Folstein MF , Folstein SE , McHugh PR (1975) “Mini-mental state”. A practical method for grading the cognitive state of patients for the clinician. J Psychiatr Res 12, 189–198. 1202204 10.1016/0022-3956(75)90026-6

[24] Franzen S , European Consortium on Cross-Cultural Neuropsychology (ECCroN), Watermeyer TJ , Pomati S , Papma J M , Nielsen TR , Narme P , Mukadam N , Lozano-Ruiz Á , Ibanez-Casas I , Goudsmit M , Fasfous A , Daugherty JC , Canevelli M , Calia C , Esther van den Berg E , Bekkhus-Wetterberg P (2022) Cross-cultural neuropsychological assessment inEurope: Position statement of the European Consortium on Cross-Cultural Neuropsychology (ECCroN). ClinNeuropsychol 36, 546–557. 10.1080/13854046.2021.1981456 34612169

[25] Franzen S , Papma JM , Van den Berg E , Nielsen TR (2021) Cross-cultural neuropsychological assessment in the European Union: A Delphi expert study. Arch Clin Neuropsychol 36, 815–830. 33043958 10.1093/arclin/acaa083 PMC8292927

[26] Storey JE , Rowland JT , Basic D , Conforti DA , Dickson HG (2004) The Rowland Universal Dementia Assessment Scale (RUDAS): A multicultural cognitive assessment scale. Int Psychogeriatr 16, 13–31. 15190994 10.1017/s1041610204000043

[27] Nielsen TR , Jørgensen K (2020) Cross-cultural dementia screening using the Rowland Universal Dementia Assessment Scale: A systematic review and meta-analysis. Int Psychogeriatr 32, 1031–1044. 32146910 10.1017/S1041610220000344

[28] Nielsen TR , Segers K , Vanderaspoilden V , Bekkhus-Wetterberg P , Minthon L , Pissiota A , Hanevold Bjørkløf G , Beinhoff U , Tsolaki M , Gkioka M , Waldemar G (2018) Performance of middle-aged and elderly European minority and majority populations on a Cross-Cultural Neuropsychological Test Battery (CNTB). Clin Neuropsychol 32, 1411–1430. 29364089 10.1080/13854046.2018.1430256

[29] Nielsen TR , Segers K , Vanderaspoilden V , Beinhoff U , Minthon L , Pissiota A , Bekkhus-Wetterberg P , Hanevold Bjørkløf G , Tsolaki M , Gkioka M , Waldemar G (2019) Validation of a European Cross-Cultural Neuropsychological Test Battery (CNTB) for evaluation of dementia. Int J Geriatr Psychiatry 34, 144–152. 30246268 10.1002/gps.5002

[30] Goudsmit M , Uysal-Bozkir Ö , Parlevliet JL , van Campen JPCM , de Rooij SE , Schmand B (2017) The Cross-Cultural Dementia Screening (CCD): A new neuropsychological screening instrument for dementia in elderly immigrants. J Clin Exp Neuropsychol 39, 163–172. 27501011 10.1080/13803395.2016.1209464

[31] Nielsen TR , Segers K , Vanderaspoilden V , Beinhoff U , Minthon L , Pissiota A , Bekkhus-Wetterberg P , Hanevold Bjørkløf G , Tsolaki M , Gkioka M , Waldemar G (2019) Validation of a brief Multicultural Cognitive Examination (MCE) for evaluation of dementia. Int J Geriatr Psychiatry 34, 982–989. 30901493 10.1002/gps.5099

[32] Nielsen TR , Vogel A , Waldemar G (2012) Comparison of performance on three neuropsychological tests in healthy Turkish immigrants and Danish elderly. Int Psychogeriatr 24, 1515–1521. 22717281 10.1017/S1041610212000440

[33] Strauss E , Sherman EMS , Spreen O (2006) A Compendium of Neuropsychological Tests. Administration, Norms, and Commentary. 3rd ed. Oxford University Press, New York.

[34] Schmidtke K , Olbrich S (2007) The Clock Reading Test: Validation of an instrument for the diagnosis of dementia and disorders of visuo-spatial cognition. Int Psychogeriatr 19, 307–321. 17147844 10.1017/S104161020600456X

[35] National Board of Health and Welfare (2017) National Guidelines for Care in Cases of Dementia –Support for Governing and Management, The National Board of Health and Welfare, Stockholm.

[36] Shulman KI (2000) Clock-drawing: Is it the ideal cognitive screening test? Geriatr Psychiatry 15, 548–561. 10.1002/1099-1166(200006)15:6<548::aid-gps242>3.0.co;2-u 10861923

[37] Pfeffer RI , Kurosaki TT , Harrah CH Jr , Chance JM , Filos S (1982) Measurement of functional activities in older adults in the community. J Gerontol 37, 323–329. 7069156 10.1093/geronj/37.3.323

[38] Gottfries GG , Noltorp S , Nørgaard N (1997) Experience with a Swedish version of the Geriatric Depression Scale in primary care centres. Int J Geriatr Psychiatry 12, 1029–1034. 9395935 10.1002/(sici)1099-1166(199710)12:10<1029::aid-gps683>3.0.co;2-d

[39] Wild D , Grove A , Martin M , Eremenco S , McElroy S , Verjee-Lorenz A , Erikson P (2005) Principles of good practice for the translation and cultural adaptation process for patient-reported outcomes (PRO) measures: Report of the ISPOR Task Force for Translation and Cultural Adaptation. Value Health 8, 94–104. 15804318 10.1111/j.1524-4733.2005.04054.x

[40] World Health Organization (1992) The ICD-10 Classification of Mental and Behavioural Disorders: Clinical Descriptions and Diagnostic Guidelines. World Health Organization, Geneva.

[41] McKeith IG , Boeve BF , Dickson DW , Halliday G , Taylor JP , Weintraub D , Aarsland D , Galvin J , Attems J , Ballard CG , Bayston A , Beach TG , Blanc F , Bohnen N , Bonanni L , Bras J , Brundin P , Burn D , Chen-Plotkin A , Duda JE , El-Agnaf O , Feldman H , Ferman TJ , Ffytche D , Fujishiro H , Galasko D , Goldman JG , Gomperts SN , Graff-Radford NR , Honig LS , Iranzo A , Kantarci K , Kaufer D , Kukull W , Lee VMY , Leverenz JB , Lewis S , Lippa C , Lunde A , Masellis M , Masliah E , McLean P , Mollenhauer B , Montine TJ , Moreno E , Mori E , Murray M , O’Brien JT , Orimo S , Postuma RB , Ramaswamy S , Ross OA , Salmon DP , Singleton A , Taylor A , Thomas A , Tiraboschi P , Toledo JB , Trojanowski JQ , Tsuang D , Walker Z , Yamada M , Kosaka K (2017) Diagnosis and management of dementia with Lewy bodies. Neurology 89, 88–100. 28592453 10.1212/WNL.0000000000004058 PMC5496518

[42] Metz CE (1978) Basic principles of ROC analysis. Semin Nucl Med 8, 283–298. 112681 10.1016/s0001-2998(78)80014-2

[43] Taber KS (2018) The use of Cronbach’s alpha when developing and reporting research instruments in science education. Res Sci Educ 48, 1273–1296.

[44] Araujo NB , Nielsen TR , Barca ML , Engedal K , Marinho V , Deslandes AC , Coutinho ES , Laks J (2020) Brazilian version of the European Cross-Cultural Neuropsychological Test Battery (CNTB-BR): Diagnostic accuracy across schooling levels. Braz J Psychiatry 42, 286–294. 32130401 10.1590/1516-4446-2019-0539 PMC7236160

[45] Salmon DP , Bondi MW (2009) Neuropsychological assessment of dementia. Annu Rev Psychol 60, 257–282. 18616392 10.1146/annurev.psych.57.102904.190024 PMC2864104

[46] Maestri G , Nicotra A , Pomati S , Canevelli M , Pantoni L , Cova I (2023) Cultural influence on clock drawing test: A systematic review. J Int Neuropsychol Soc 29, 704–714. 36426579 10.1017/S1355617722000662

[47] Tombaugh TN , McIntyre NJ (1992) The mini-mental state examination: A comprehensive review. J Am Geriatr Soc 40, 922–935. 1512391 10.1111/j.1532-5415.1992.tb01992.x

[48] Arentoft A , Byrd D , Robbins RN , Monzones J , Miranda C , Rosario A , Coulehan K , Fuentes A , Germano KK , D’Aquila E , Sheynin J , Fraser F , Morgello S , Rivera Mindt M (2012) Multidimensional effects ofacculturation on English-language neuropsychological testperformance among HIV+ Caribbean Latinas/os. J Clin ExpNeuropsychol 34, 814–825. 10.1080/13803395.2012.683856 PMC3434263 22624844

[49] Boone KB , Victor TL , Wen J , Razani J , Pontón M (2007) Theassociation between neuropsychological scores and ethnicity,language, and acculturation variables in a large patient population. Arch Clin Neuropsychol 22, 355–365. 17320344 10.1016/j.acn.2007.01.010

[50] Razani J , Burciaga J , Madore M , Wong J (2007) Effects ofacculturation on tests of attention and information processing in anethnically diverse group. Arch Clin Neuropsychol 22, 333–341. 17298874 10.1016/j.acn.2007.01.008

[51] Al-Jawahiri F , Nielsen TR (2021) Effects of acculturation on the Cross-Cultural Neuropsychological Test Battery (CNTB) in a culturally and linguistically diverse population in Denmark. Arch Clin Neuropsychol 36, 381–393. 31942602 10.1093/arclin/acz083

[52] Statistics Sweden (2022) Foreign-born by country of birth, sex and year of immigration 31 December 2021. https://scb.se/en/finding-statistics/statistics-by-subjectarea/population/population-composition/populationstatistics/Last updated February 22, 2022, Accessed on March 3, 2023.

[53] Majlesi AR , Plejert C (2018) Embodiment in tests of cognitive functioning: A study of an interpreter-mediated dementia evaluation. Dementia 17, 138–163. 26924841 10.1177/1471301216635341

[54] Morris JC (1997) Clinical dementia rating: A reliable and valid diagnostic and staging measure for dementia of the Alzheimer type. {Int Psychogeriatr 9, 173–176. 9447441 10.1017/s1041610297004870

