# Supplementary material for: Diagnostic Accuracy of the Swedish Version of the Multicultural Cognitive Examination for Cognitive Assessment in Swedish Memory Clinics
Source: J Alzheimers Dis. 2024 Jan 16;97(2):715–26. doi: 10.3233/JAD-230998 (PMC10836550; doi:10.3233/JAD-230998)
Supplement: Supplementary Material [file jad-97-jad230998-s001.pdf]

Supplementary Material

Diagnostic Accuracy of the Swedish Version of the Multicultural Cognitive Examination for Cognitive Assessment in Swedish Memory Clinics

Cognitive scores on the MCE-S in patients with SCI, MCI without AD pathology, MCI with AD pathology, dementia, and other diagnoses.

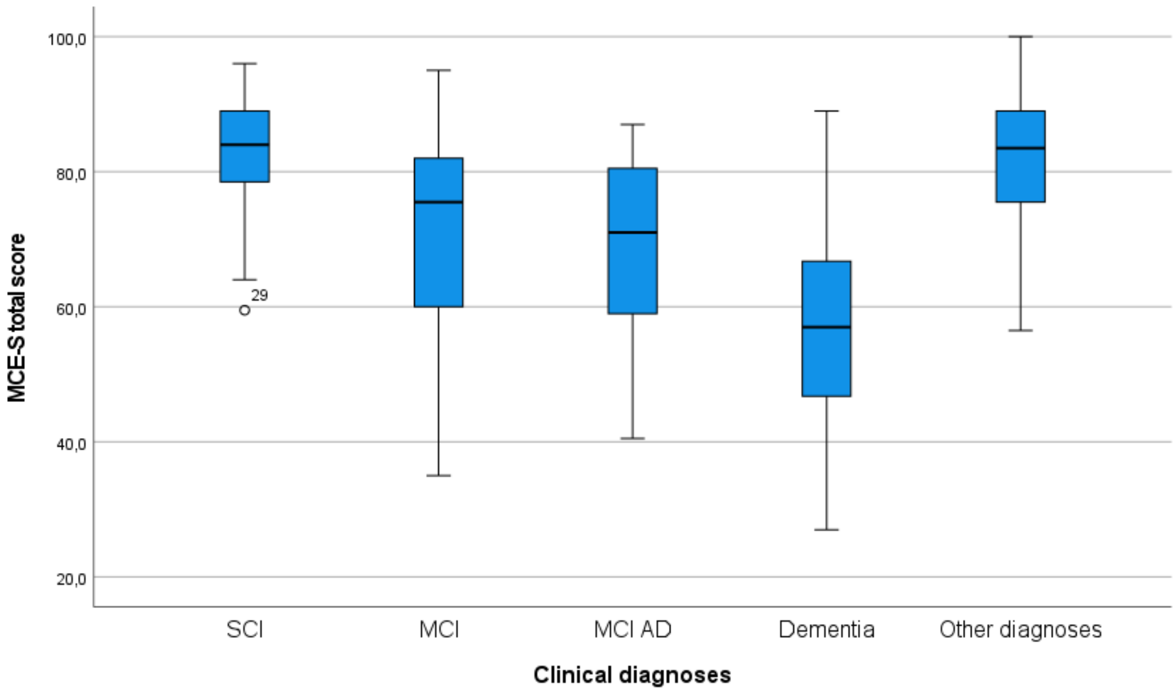

The MCE-S scores decreased with increasing cognitive impairment. Patients with MCI with proven AD pathology tended to have lower MCE-S scores than did those with MCI without AD.
